# Changes in Porous Parameters of the Ion Exchanged X Zeolite and Their Effect on CO_2_ Adsorption

**DOI:** 10.3390/molecules26247520

**Published:** 2021-12-11

**Authors:** Andżelika Gęsikiewicz-Puchalska, Michal Zgrzebnicki, Beata Michalkiewicz, Agnieszka Kałamaga, Urszula Narkiewicz, Antoni W. Morawski, Rafal Wrobel

**Affiliations:** 1 Department of Catalytic and Sorbent Materials Engineering, Faculty of Chemical Technology and Engineering, West Pomeranian University of Technology, Piastów Ave. 42, 71-065 Szczecin, Poland; andzelika.gesikiewicz@zut.edu.pl (A.G.-P.); michal.zgrzebnicki@zut.edu.pl (M.Z.); Beata.Michalkiewicz@zut.edu.pl (B.M.); agnieszka.kalamaga@zut.edu.pl (A.K.); 2 Department of Inorganic Chemical Technology and Environment Engineering, Faculty of Chemical Technology and Engineering, West Pomeranian University of Technology, Piastów Ave. 42, 71-065 Szczecin, Poland; urszula.narkiewicz@zut.edu.pl (U.N.); antoni.morawski@zut.edu.pl (A.W.M.)

**Keywords:** CO_2_ adsorption, selectivity, zeolite 13X, ion exchange, CCS

## Abstract

Zeolite 13X (NaX) was modified through ion-exchange with alkali and alkaline earth metal cations. The degree of ion exchange was thoroughly characterized with ICP, EDS and XRF methods. The new method of EDS data evaluation for zeolites was presented. It delivers the same reliable results as more complicated, expensive, time consuming and hazardous ICP approach. The highest adsorption capacities at 273 K and 0.95 bar were achieved for materials containing the alkali metals in the following order K < Na < Li, respectively, 4.54, 5.55 and 5.94 mmol/g. It was found that it is associated with the porous parameters of the ion-exchanged samples. The Li_0.61_Na_0.39_X form of zeolite exhibited the highest specific surface area of 624 m^2^/g and micropore volume of 0.35 cm^3^/g compared to sodium form 569 m^2^/g and 0.30 cm^3^/g, respectively. The increase of CO_2_ uptake is not related with deterioration of CO_2_ selectivity. At room temperature, the CO_2_ vs. N_2_ selectivity remains at a very high stable level prior and after ion exchange in co-adsorption process (X_CO2_ during adsorption 0.15; X_CO2_ during desorption 0.95) within measurement uncertainty. Additionally, the Li_0.61_Na_0.39_X sample was proven to be stable in the aging adsorption-desorption tests (200 sorption-desorption cycles; circa 11 days of continuous process) exhibiting the CO_2_ uptake decrease of about 6%. The exchange with alkaline earth metals (Mg, Ca) led to a significant decrease of SSA and micropore volume which correlated with lower CO_2_ adsorption capacities. Interestingly, the divalent cations cause formation of mesopores, due to the relaxation of lattice strains.

## 1. Introduction

The emission of carbon dioxide into the atmosphere from fossil fuel combustion is considered as a major source of intensifying of the greenhouse effect [1,2]. Although anthropogenic methane is a more potent greenhouse gas than CO_2_, its lower emission provides the second-largest contribution to global warming [3,4]. The growing awareness of the international community with regard to climate change has led to search for technologies designed to lessen greenhouse gas emission. The extensive discussion about complex impact of anthropogenic CO_2_ on environment, descriptions of major threats, critical counterarguments, etc. were extensively described elsewhere [1]. Nowadays, a few general methods are being used to CO_2_ capture from flue gas, including absorption, adsorption, cryogenic processes and membrane separation [5,6,7,8]. Adsorption with solid sorbents followed by underground storage, so-called carbon capture and storage (CCS), is one of the most promising options to reduce CO_2_ emission [9]. Different types of porous materials, such as activated carbons, molecular sieves, metal-organic frameworks and zeolites, could be used as CO_2_ adsorbents [10,11,12,13].

Zeolites are microporous crystalline materials composed of SiO_4_ and AlO_4_ tetrahedra connected by oxygen atoms. The negative charge in zeolite framework is balanced by exchangeable cations [14,15]. Due to well defined pores and high selectivity, zeolites are suitable materials for CO_2_ capture. A number of papers concerning CO_2_ adsorption on zeolite 13X have been published previously. Harlick and Tezel [16] measured CO_2_ adsorption isotherms on zeolites with different silica-to-aluminum ratios, including 13X, NaY, HiSiv-1000, HY-5, ZSM-5-30 and HiSiv-3000. The highest adsorption capacities were found with zeolite 13X attaining 4,6 mmol/g (295 K, 1 bar). This was attributed to the low Si/Al ratio in 13X zeolite framework. Siriwardane et al. [17] investigated the CO_2_ adsorption on five type of zeolites such as 4A, 5A, 13X, APG-II and WE-G 592. Zeolite 13X showed the highest adsorption capacity at 5.3 mmol/g (303 K, 20 bar) and 4.3 mmol/g (393 K, 20 bar). According to the authors it was associated with the largest pore diameter and lowest Si/Al ratio of zeolite 13X among of studied samples. Sarker et al. [18] examined the CO_2_ adsorption on zeolite 13X at a temperature range of 293–333 K and pressure up to 35 bar. The adsorption capacities were 7.0 and 5.2 mmol/g at 293 and 333 K, respectively. V. Garshasbi et al. [19] studied the adsorption equilibrium of CO_2_ on zeolite 13X prepared from natural clays such as kaolin, bentonite and feldspath. The CO_2_ uptake capacity of the zeolite 13X obtained from kaolin reached 6.9 mmol/g (298 K, 20 bar). Cavenati et al. [20] investigated the isosteric heat of adsorption of carbon dioxide, nitrogen and methane on zeolite 13X. CO_2_ showed higher heat of adsorption (37.2 kJ/mol) than N_2_ (12.8 kJ/mol) and CH_4_ (15.3 kJ/mol). The authors concluded that the preferential adsorption capacity of CO_2_ on zeolite 13X makes it a good candidate for CO_2_ sequestration from flue gas. Walton et al. [21] examined the CO_2_ adsorption equilibrium isotherms on the alkali metal ion-exchanged X and Y samples. The authors observed that the CO_2_ uptake increase with decreasing cation size for both zeolites. As a result, zeolite X and Y, which contain small Li cations give the highest CO_2_ capacity of 5.6 and 5.2 mmol/g (298 K, 1 bar), respectively. According to the authors, it is associated with greater ion-quadrupole interactions with CO_2_ of these samples than materials comprising lager cations. It is well-known that this ion-quadrupole interactions play a significant role in the CO_2_ adsorption mechanism of zeolite adsorbents [22]. However, the available pore volume of zeolite 13X and its ion-exchanged forms is another factor which can influence CO_2_ adsorption.

This paper contains detailed characteristic of zeolite 13X and its ion-exchanged forms with an analysis of crucial for CO_2_ adsorption micropores below diameter of 0.8 nm. The ion-exchanged form of zeolite 13X were thoroughly characterized with ICP, XRF and modified EDS methods. There is proposed a new EDS characterization approach of cation exchange degree specific for zeolites. This approach was proven to be consistent with ICP results and free of ICP methods’ drawbacks, such as hazardous acid treatment, high cost, destruction of the sample, etc.

In order to test the stability of the obtained zeolites, we have performed 200 cycles of CO_2_ adsorption/desorption, which took about 11 days. To the best of our knowledge, there are no reports in the literature about stability tests in multiple adsorption-desorption cycles of zeolite 13X and its ion-exchanged forms. Stability of adsorbents is very important for its industry application. Moreover, we have also measured selectivity during competitive co-adsorption of N_2_ and CO_2_ with a mass spectrometer. Such an approach is rare in the literature because of the complexity of the measurements and most studies provide apparent selectivity, i.e., a comparison of adsorption uptakes of pure gases, such as N_2_ and CO_2_ [23,24].

## 2. Results

**Figure 1:**
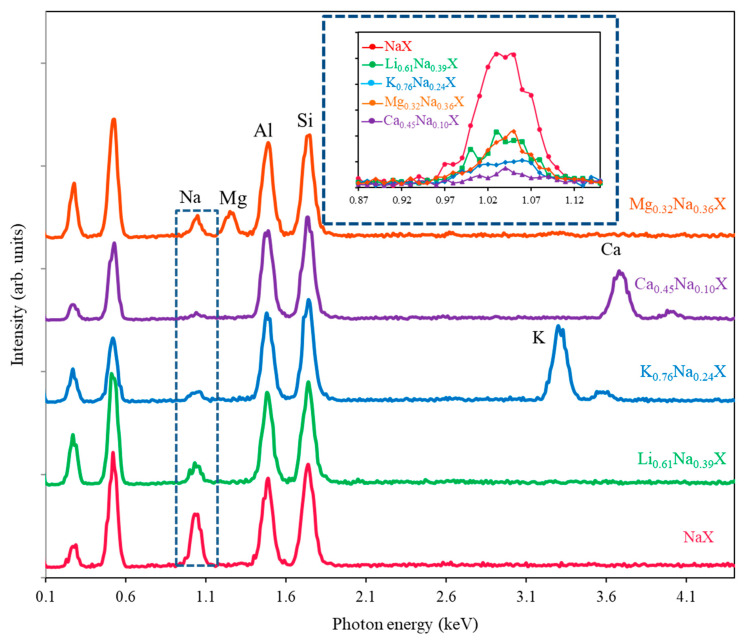
EDS spectra of the studied zeolite samples. Inset presents changes of the Na signal.

Through ion exchange, one can obtain the substitution of sodium cations by other ions in the framework of zeolite. However, the degree of exchange is dependent on the equilibrium between the solution and solid. Thus, the full substitution of sodium cations is not feasible in simple ion exchange from a solution. Therefore, in order to measure the effectiveness of such an exchange, EDS measurements were performed. This method is highly efficient for heavy elements, but it has some limitations for light elements. For instance, older EDS detectors were not able to detect lithium using standard configuration. This has changed a few years back, when new windowless detectors were developed [25]. Nevertheless, we have performed EDS analysis on an older device, which was simply not designed to detect lighter elements than boron. Therefore, to tackle this issue, one may measure the effectiveness of substitution by monitoring the concentration of sodium. The initial value of sodium is well-defined in 13X zeolite. Another problem is stability of signal and roughness of the sample. Both these factors affect quantitative analysis. However, in case of 13X zeolites, the atomic concentration of silicon and aluminum is constant, and thus, this may serve as an internal standard. Due to the preservation of charge the removed sodium cations have to be replaced by other mono- or bivalent cations of equal charge. Figure 1 presents the EDS spectra of all samples normalized to the silicon signal.

The sodium signals are presented in the inset in Figure 1. Notice that lithium gives no EDS signal. The zeolite composition of anions does not change during the cation exchange process. Normalization with respect to the silicon signal solves the problem of the electron beam current change during the measurements. After normalization, one can compare the intensity of sodium signals in samples (Figure 1 inset). The intensity of the initial sample (NaX) may be assumed as 100%, and thus, the remaining sodium in the other samples may be evaluated. The introduced ions replace the sodium ions with preservation of charge, e.g., one magnesium ion replaces two sodium ions. One should underline that monitoring of depletion of sodium ions enables the measurement of lithium ions, which are not detectable via the EDS method. The quantitative measurement of zeolite ion exchange with EDS is far easier compared to ICP because the samples do not have to be dissolved in fluoric acid. The EDS results were compared with the ones obtained with ICP method, which is commonly considered as very reliable. Moreover, the XRF analysis was also applied with the same internal standard approach, as it was in the case of EDS. The XRF method has few advantages over ICP and EDS, e.g., it is inexpensive, does not require vacuum conditions and does not destroy the sample.

In Table 1, the content of sodium is given as obtained with three different methods. The areas of sodium signals in both the EDS and XRF methods were calculated numerically with the trapezoidal rule.

One may notice that three methods yield the same results within the measurement uncertainty. It can be noticed that despite three ion exchange cycles, there are still sodium ions in all samples. The most successful ion exchange was obtained for calcium cations and the least successful for lithium ones. In order to obtain higher ion exchange degrees, more cycles may be applied. An alternative solution may be the application of column packed with NaX and passing a feed solution containing proper cation (Li^+^, K^+^, Mg^2+^, Ca^2+^), which is much more efficient. Conversely, the method applied in this research compared to column exchange is very simple and results in homogeneous product.

**Figure 2:**
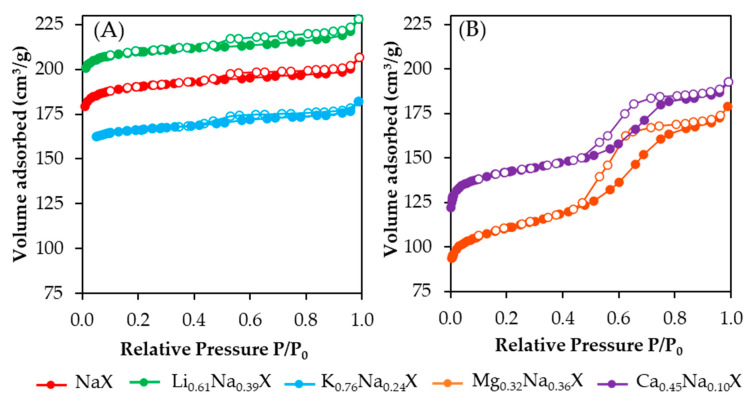
N_2_ adsorption-desorption isotherms of zeolite samples containing the monovalent (**A**) and divalent cations (**B**). Full and empty circles denote the adsorption and desorption process, respectively.

Figure 2 presents nitrogen adsorption-desorption isotherms of cation exchanged samples. One can observe significant differences between the shape of the isotherms obtained for zeolites containing monovalent and divalent cations. The isotherms of zeolite samples comprised of alkali metals (Li, Na, K) are combination of types Ib and IVa according to the IUPAC classification. For low relative pressure the N_2_ adsorption increased very fast with the increase of P/P_0_, which is characteristic for microporous materials. In addition, the isotherms exhibited hysteresis loop for P/P_0_ > 0.4, which indicated the presence of mesopores. Axes in both Figure 2A,B were set with the same range of values for better visualization of shape differences. 

**Figure 3:**
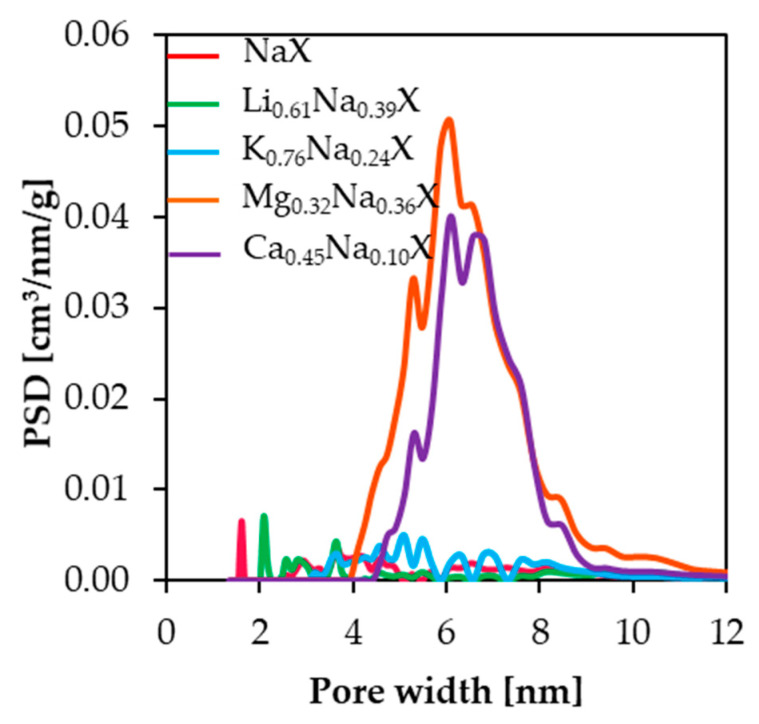
Pore size distributions of studied samples calculated from N_2_ adsorption/desorption isotherms at 77 K.

In case of adsorbents containing alkaline earth metals (Ca, Mg), the isotherm shape moved toward a type IVa isotherm, which is associated with greater quantity of mesopores fraction. Moreover, a more pronounced hysteresis phenomenon is the result of a significantly increased volume of mesopores above a diameter of 4 nm (confirmed by Figure 3).

Table 2 shows the textural parameters of all the samples obtained with the BET and DFT methods. The highest specific surface area and micropore volume was achieved for Li_0.61_Na_0.39_X sample 624 m^2^/g and 0.35 cm^3^/g, respectively. In case of the ion exchanged forms containing the metal cations with higher molecular weight (K, Mg, Ca), significant reductions of SSA and micropore volume were observed. 

Based on N_2_ adsorption-desorption isotherms measurements at 77 K, the pore size distributions were determined. The PSD results of the studied samples were estimated by applying the DFT theory and are presented in Figure 3. Mesopore volume presented in Table 2 was calculated by subtracting values of cumulative pore volume at 2 nm from volume at 12 nm; therefore, V_meso_ indicates volume of mesopores in specific range of pore width. The samples, which contain small cations, such as lithium and sodium, do not exhibit significant amount of mesopore volume, i.e., in the range 2–50 nm. These samples have the distinct peaks in the range of 2 nm, which is associated with the presence of micropores fraction.

For zeolite samples comprising divalent cations, significant increases of pore volume in the range of 4.0–12.0 nm were observed, which indicates that the introduction of the divalent cations into the zeolite framework leads to formation of the mesopores. 

**Figure 4:**
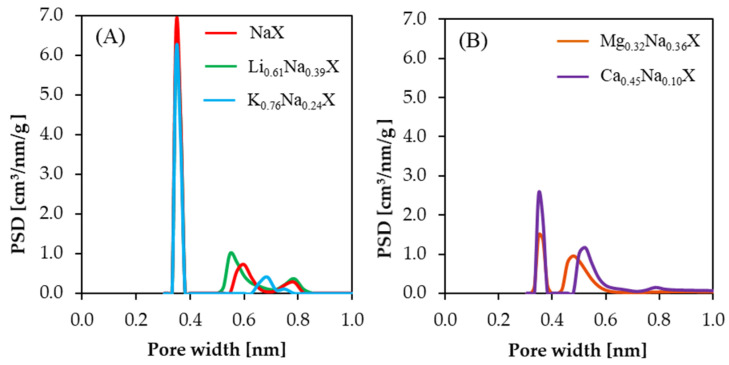
Pore size distributions of samples containing the monovalent (**A**) and divalent cations (**B**), calculated from CO_2_ isotherms at 273 K.

One has to note that the N_2_ adsorption-desorption isotherms method allows to estimate the micropore volume in the range of 1.2–2.0 nm [26] and is “blind” to micropores smaller than 1.2 nm. Therefore, pores smaller than 1.2 nm are not observed in Figure 3, although they do exist in all samples. Pores with diameter below 1 nm were determined from CO_2_ adsorption isotherms measurement at 273 K. The PSDs results of investigated samples are presented in Figure 4.

In case of samples consisting of monovalent cations, subtle changes of pore volume in the range of 0.30–0.45 nm were observed. However, pore volume in the range of 0.50–0.80 nm increased in the sequence of Li > Na > K. The exact values of submicropore volumes are summarized in Table 2. It is expected that the increase of volume of pores smaller than 1 nm for Li_0.61_Na_0.39_X sample leads to higher CO_2_ uptake. 

The samples composed of divalent cations indicated a significant reduction of pore volume in the range of 0.35–0.40 nm. It can be deduced that the presence of Ca^2+^ and Mg^2+^ cations in zeolite framework leads to blockage of the CO_2_ access to small submicropores or to collapse of submicropores.

**Figure 5:**
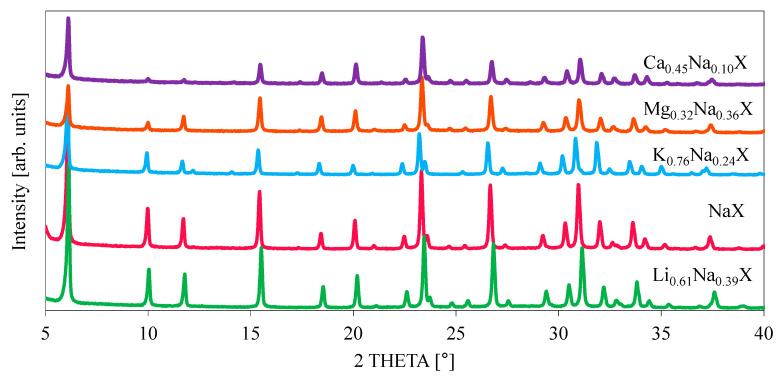
Diffraction patterns of investigated zeolite samples.

**Figure 6:**
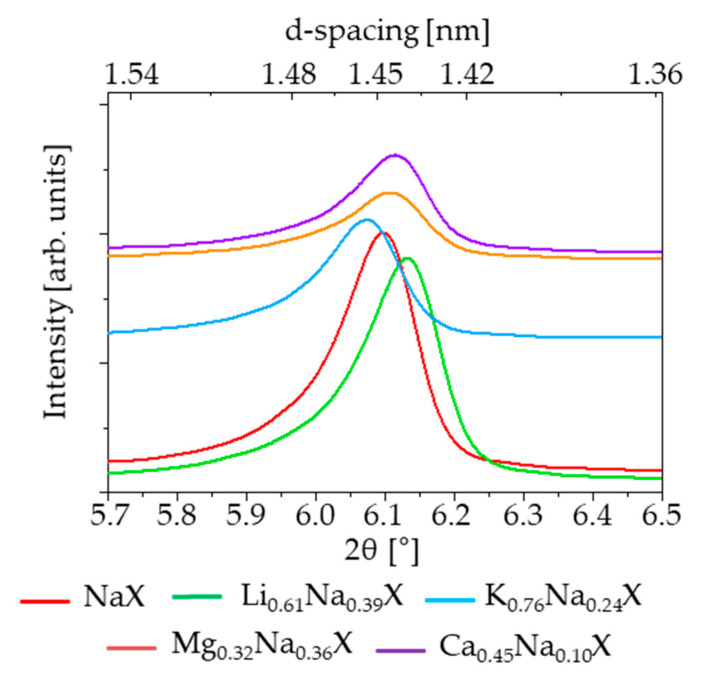
The (111) XRD reflexes of the sorbents after ion-exchange.

The crystallinity of the zeolite samples, before and after cation exchange treatment, was examined by X-ray diffraction. The diffractograms of investigated samples are illustrated in Figure 5 and Figure 6. It can be observed that all the modified samples exhibit the typical peak patterns of the faujasite (FAU) structure [27,28], which is characteristic for the zeolite X and Y. However, the intensities of the diffraction peaks for K_0.76_Na_0.24_X, Mg_0.32_Na_0.36_X and Ca_0.45_Na_0.10_X samples were significantly reduced. For all diffractograms the zeolite X phase was attributed to pattern 00-038-0237.

Focusing on single XRD peak, the signal shifts towards lower theta angles with increasing radius of monovalent cations. It is related with an increase of lattice constant of zeolite with increase of radius of introduced cation. The values of cation ionic radius and lattice parameters are presented in Table 3.

The lattice constants were evaluated based on (111) reflex of zeolite 13X (pdf4+ card 00-038-0237 sodium form of zeolite 13X). According to this card the lattice constant is 24.99 Å, which is comparable to our measurement. In case of samples which consist of divalent cations, the effect is more complex, because two monovalent ions in the zeolite framework are replaced by a single divalent ion.

The ion exchange of sodium to divalent cations leads to formation of significant amount of mesopores in the size range 2–12 nm (Figure 3). In case of monovalent cations this pore volume is negligible and amount about 0.01 cm^3^/g. After ion exchange this volume increases one order of magnitude and amounts 0.12 and 0.08 cm^3^/g for magnesium and calcium form of zeolite, respectively. The experimental confirmation of presence of such mesopores is reliable; however, the mechanism of their formation is not clear. To address this issue, the lattice strains were measured. It was hypothesized that ion exchange may lead to induction of lattice strains and in case of very high values of strains the crystallites may undergo relaxation by cracking, and thus, mesopores might be formed. To evaluate the value of strains the Wilson formula [29], Equation (1) was applied:

(1) $$\varepsilon =\frac{B}{4tan\theta }$$

where ε is the lattice strain and *B* is the difference in reflection breadth of standard and analyzed sample.

The presence of strain causes broadening of XRD reflections. The measure of broadening is so called integral breadth, i.e., area of reflection divided by its height. The strain was evaluated based on the most intense reflection (111). The XRD apparatus effect also contributes to broadening; therefore, this phenomenon should be corrected by the application of strain-free standard. For the purpose of this work, the sodium form of zeolite, i.e., NaX, was considered as a standard. Therefore, the obtained values of the strain are relative to NaX. Positive values denote higher strains compared to NaX and negative ones denote smaller strains. In Table 4 are presented values of integral breadth and the calculated lattice strains. One may notice that the introduction of anything other than sodium ions causes an increase of lattice strains. The highest values of strain are observed to magnesium and calcium forms of zeolite, proving the concept that the formation of mesopores is a result of strain relaxation. Moreover, the highest lattice strain is observed for magnesium form of zeolite, which exhibits the highest volume of mesopores. Despite the higher ionic radius of calcium, the strains are smaller compared to magnesium form of zeolite. This may be explained by notion that strains are higher in case of mixture of ions of different radii in the zeolite lattice. This is due to a variation of d-spacing caused by different cations. Therefore, it is expected to have smaller values of lattice strain in the case of homogeneous or nearly-homogeneous materials. Indeed, the sodium form exhibits the smallest values of strain and the calcium form, which contains only 10% of sodium atoms and has smaller strains compared to the magnesium form.

**Figure 7:**
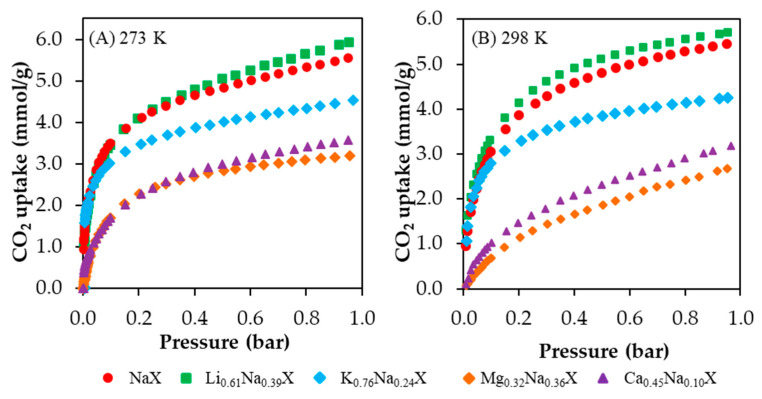
CO_2_ adsorption isotherms at 273 K (**A**) and 298 K (**B**) of cation exchanged samples.

CO_2_ adsorption isotherms obtained at 273 K and 298 K are presented in Figure 7A,B, respectively. The data values of CO_2_ adsorption capacity are shown in Table 5. The CO_2_ uptake increased in the order of Mg^2+^ < Ca^2+^ < K^+^ < Na^+^ < Li^+^. One should notice that the obtained results are positively correlated with the increased micro- and submicropore volume of these samples.

**Figure 8:**
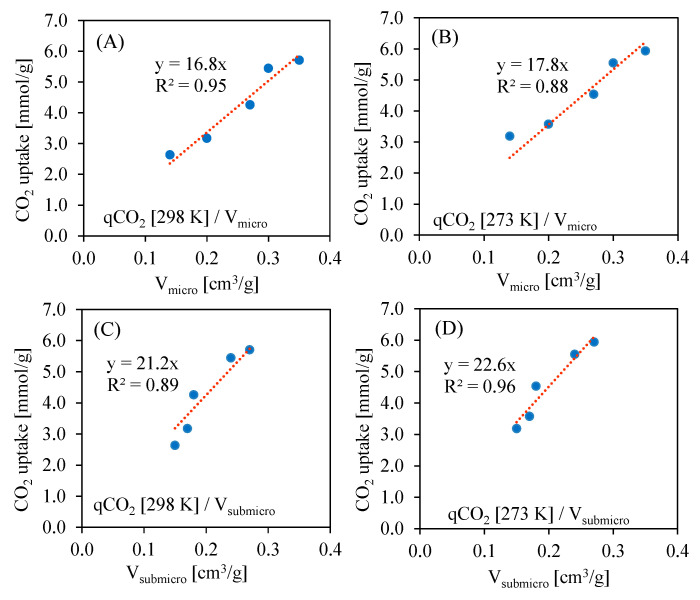
Correlations of CO_2_ uptake at 0.95 bar vs. micropore (**A**,**B**) and submicropore (**C**,**D**) volumes at 298 K and 273 K.

Figure 8 presents the correlations of CO_2_ uptake vs. micropore or submicropore volumes at 273 K and 298 K. As the CO_2_ uptake is negligible for non-porous materials, the linear equation has only one parameter, i.e., slope. In all cases, the R^2^ parameter is above 0.9, which may indicate a high impact of the pore volume on the CO_2_ uptake. One should notice that in case of submicropores, the slope has a higher value compared to impact. This indicates that submicropores are crucial for CO_2_ sorption, which is supported by other studies [30,31,32]. 

The Li_0.61_Na_0.39_X form of zeolite indicated the CO_2_ adsorption capacity of 5.94 mmol/g and 5.71 mmol/g at 273 K and 298 K, respectively. The other forms of ion-exchanged samples showed a decrease of CO_2_ uptake compared with the sodium form. It is worth to notice that Li_0.61_Na_0.39_X and NaX samples indicated the rapid increase of CO_2_ adsorption at a low equilibrium pressure. The same effect was observed for the potassium-exchanged form. Nevertheless, in case of the K_0.76_Na_0.24_X sample, this tendency declines at a higher pressure range. This is related to a reduction of the micropores fraction. 

Moreover, sorbent materials might be characterized in terms of normalized CO_2_ uptake, which in our case was obtained by dividing the CO_2_ uptake at 273 K by S_BET_. The following values were obtained: 9.52 × 10^−3^ mmol/m^2^ for Li_0.61_Na_0.39_X, 9.75 × 10^−3^ mmol/m^2^ for NaX, 9.15 × 10^−3^ mmol/m^2^ for K_0.76_Na_0.24_X, 9.33 × 10^−3^ mmol/m^2^ for Mg_0.32_Na_0.36_X and 8.33 × 10^−3^ mmol/m^2^ for Ca_0.45_Na_0.10_X. These values might provide useful information, mostly about the influence of the surface chemistry or some additional interactions. As can be seen, sample NaX has the highest normalized adsorption capacity, and for sample Li_0.61_Na_0.39_X, this value is slightly lower. Interestingly, it might be concluded that the higher the amount of sodium in the zeolite is, the higher the normalized adsorption capacity is.

**Figure 9:**
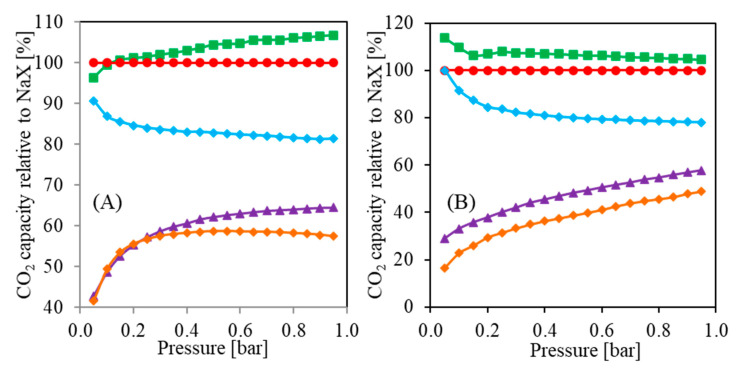
Relative capacity vs. pressure: (**A**) CO_2_ sorption at 273 K; (**B**) CO_2_ sorption at 298 K.

Figure 9 presents CO_2_ capacities relative to NaX form vs. pressure obtained based on the results from Figure 7. One can notice that the ion exchange differently affects the CO_2_ capacity dependence on CO_2_ pressure. In Figure 9A, the Ca_0.45_Na_0.10_X and Mg_0.32_Na_0.36_X are about 60% less effective at a pressure of 0.05 bar compared to NaX. At a pressure of about 0.3 bar, the difference is smaller, i.e., about 40%. Worth noting is that at a higher pressure, the Ca_0.45_Na_0.10_X form is more effective than Mg_0.32_Na_0.36_X. Interestingly, the Li_0.61_Na_0.39_X form is more effective at higher pressures, and it can be expected that even higher pressures will result in better performance compared to NaX.

On the other hand, the results presented in Figure 9B differ from the results in Figure 9A. An increase of temperature led to a change in the slope of the Li_0.61_Na_0.39_X curve. It can be expected that higher pressures will result in lower sorption capacity than NaX. Furthermore, both Ca_0.45_Na_0.10_X and Mg_0.32_Na_0.36_X curves indicate an increasing relative CO_2_ capacity in the whole presented range. Higher pressures might result in even higher carbon dioxide uptake.

**Figure 10:**
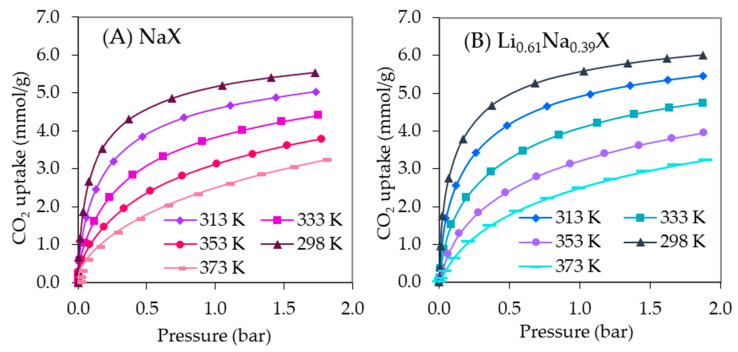
CO_2_ adsorption isotherms at different temperatures for the NaX (**A**) and Li_0.61_Na_0.39_X (**B**) samples.

Figure 10 presents the CO_2_ adsorption isotherms of NaX and Li_0.61_Na_0.39_X zeolites measured at different temperatures.

Two and three parameter adsorption models (Freundlich, Langmuir, Toth, Sips) were used to describe equilibrium data of Li_0.61_Na_0.39_X and NaX, respectively, at different temperatures. The parameters of the equations were calculated by minimizing the sum of the squares of the errors. The solver add-in with Microsoft’s spreadsheet, Excel 2013, was used for calculations. It was found that the Sips isotherms gave the highest accuracy in fitting the experimental data for Li_0.61_Na_0.39_X and NaX. 

Table 6 and Table 7 list Sips isotherm parameters for different temperatures for NaX and L_i0.61_N_a0.39_X. On the basis of *n* values, the heterogeneity of the surface of both zeolites can be assumed.

**Figure 11:**
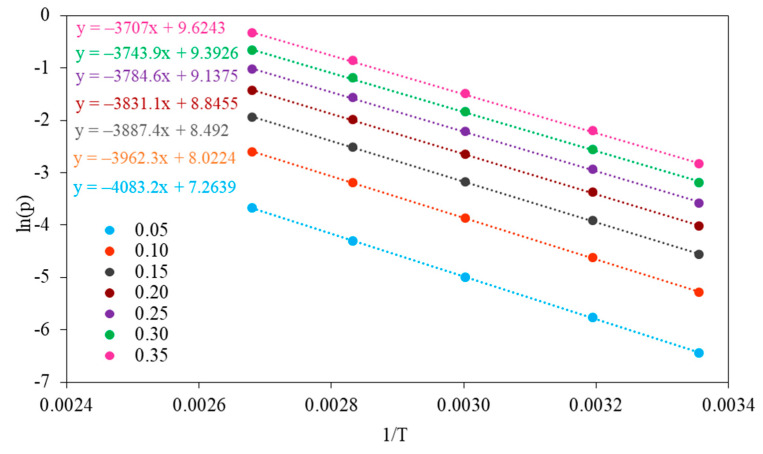
Plots of *ln(p)* vs. 1/T for surface coverage from 0.05 to 0.35 for NaX.

**Figure 12:**
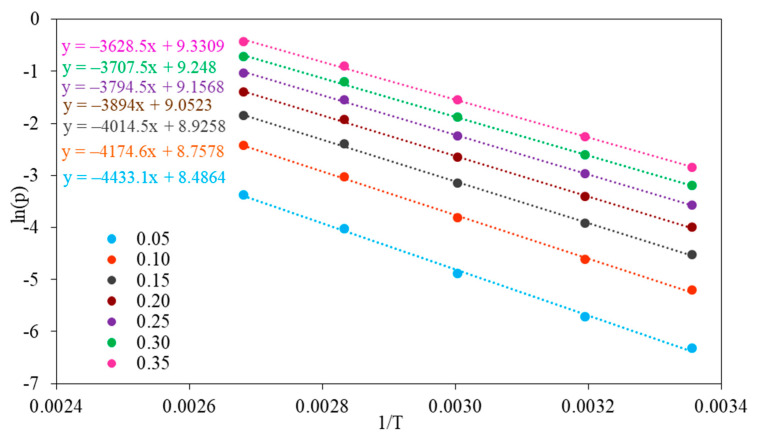
Plots of *ln(p)* vs. 1/T for surface coverage from 0.05 to 0.35 for Li_0.61_Na_0.39_X.

The isosteric heat of adsorption was estimated using the Sips equation combined with the Clausius–Clapeyron equation. The isosteric heats of adsorption were determined by evaluating the slope of the plot *ln(p)* versus 1/T at a constant coverage in the range of 0.05–0.35 (Figure 11 and Figure 12).

The isosteric heat of adsorption is the ratio of the infinitesimal change in the adsorbate enthalpy to the infinitesimal change in the amount adsorbed. The values of isosteric heat of adsorption give the strength of the interaction between the adsorbate and surface of the adsorbent.

**Figure 13:**
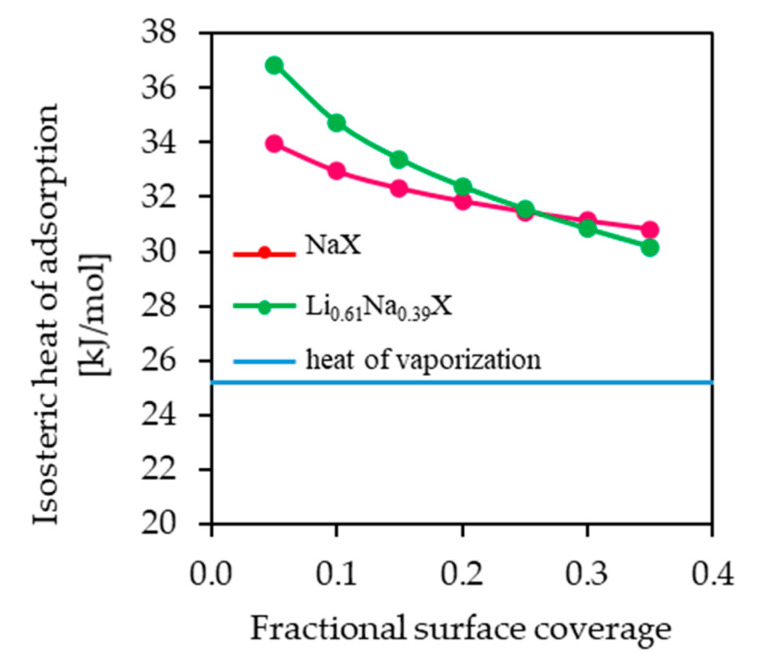
The isosteric heat of adsorption as a function of surface coverage. The horizontal line indicates the heat of vaporization at the boiling point of CO_2_ (25.16 kJ/mol).

The values of isosteric heat of adsorption for fractional surface coverage of 0.05–0.35 are similar for NaX and Li_0.61_Na_0.39_X (31–37 kJ/mol) and are much higher than heat of vaporization at the boiling point of CO_2_ (Figure 13). The values of isosteric heat of adsorption indicated the physical nature of the adsorption mechanism. 

The isosteric heat of adsorption decreased with an increase in the fractional surface coverage. The interaction between molecules of CO_2_ and NaX and Li_0.61_Na_0.39_X showed a progressive decrease with CO_2_ loading. This can be attributed to strong interactions of CO_2_ with zeolites surface. However, the adsorption places vary with respect to the heat of adsorption and the most favorable are the ones with highest adsorption energy. It can be assumed that the intermolecular interaction between CO_2_ molecules adsorbed on the zeolites surface is negligible.

A suitable adsorbent for CO_2_ capture from flue gases should exhibit, among others, good stability after repeated adsorption/desorption cycles. This is a very important attribute due to its direct impact on the economics of commercial scale applications. The high cycle life and the low price are interconnected. Therefore, the sorbents of the highest CO_2_ adsorption capacity were selected to study their stability after 200 adsorption/desorption cycles.

Aging tests were performed in a horizontal quartz tube, which was loaded with samples placed in smaller tubes enabling gas to flow through the bed. The flow of the carbon dioxide during tests was 100 cm^3^/min. The swing temperature adsorption was applied. Heating and cooling to 373 K and 303 K, respectively, was performed every 80 min and defined as one cycle. 

**Figure 14:**
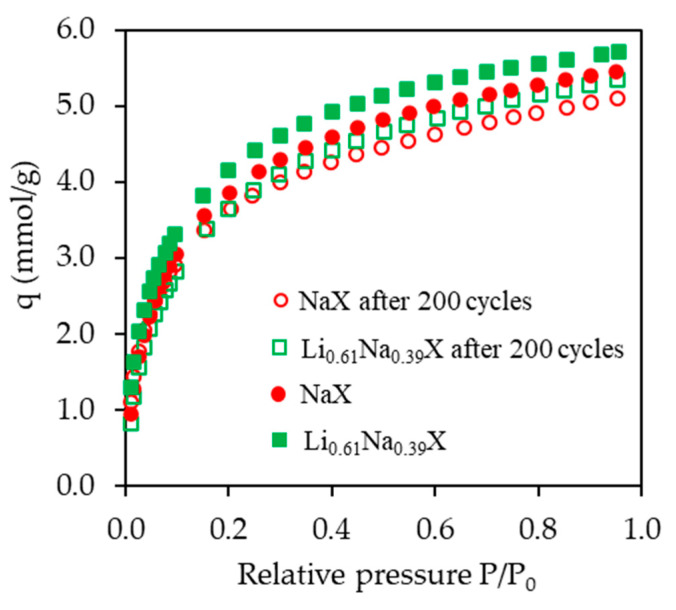
CO_2_ adsorption isotherms at 298 K of Li_0.61_Na_0.39_X and NaX samples before and after 200 adsorption/desorption aging cycles.

The obtained results are presented in Figure 14 and Table 5. It can be observed that the CO_2_ adsorption capacity after 200 cycles, i.e., after about 11 days of continuous process, decreased only by 6.5% for both tested samples. 

**Figure 15:**
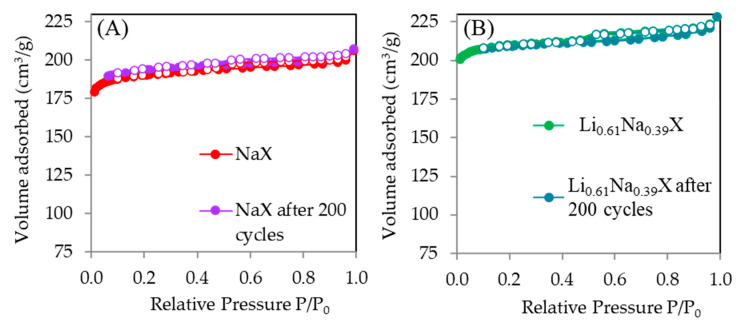
N_2_ adsorption/desorption isotherms of particular zeolites before and after aging tests: (**A**) NaX, (**B**) Li_0.61_Na_0.39_X.

The impact of aging on textural properties is presented in Table 8. The corresponding nitrogen adsorption/desorption isotherms are in Figure 15. One can notice that those results are the same within the measurement uncertainty, and thus, indicate the preserved structure of the zeolite, with the intact specific surface area and pore volumes. Therefore, one can conclude that changes in CO_2_ uptakes indicate the changes in the chemistry of the surface. Possible mechanism might be the formation of lithium and sodium carbonates after long exposure to CO_2_ and water traces [36]. 

A sorbent for CCS technology requires high CO_2_ sorption uptake connected with high selectivity toward CO_2_ in the mixture of processed gases. In the literature, very often the CO_2_ uptakes are given for pure CO_2_ atmosphere and with no selectivity or with apparent selectivity only. Apparent selectivity refers to the measurement of uptakes of pure gases and the calculation of the selectivity from these values. Such an approach may deliver unrealistic values of selectivity compared to co-adsorption conditions. The literature with values of selectivity measured in co-adsorption conditions is rare. This situation results from the far more difficult measurement procedure of selectivity in such conditions. One of the methods requires application of mass spectrometer and special procedure to obtain required repeatability. In this work, the true selectivity in co-adsorption was determined by the analysis of gases during the adsorption-desorption cycles by a mass spectrometer. A mixture of the following composition was used as an adsorption mixture—15 vol.% of CO_2_, 85 vol.% of N_2_. Helium was used as a carrier gas and argon as an internal standard. The desorption step included heating the sample to 473 K. The obtained results are presented in Table 9, while Figure S1 in Supplementary Materials presents example of raw m/z signals measured by the mass spectrometer. 

The presented CO_2_ molar fraction is the mean of seven measurements for each sample. A total of eight cycles were performed, but the first cycle was always ignored in calculations due to drying the sample. Including the results from all cycles would lead to a much higher standard deviation and would be pointless; thus, the first cycle was treated like a pretreatment before a main analysis. Data showing the difference between first and second cycle of Li_0.61_Na_0.39_X analysis was presented in Supplementary Information (S1). One can notice that there is a visible difference between peaks of m/z = 44 in Figure S1A,B.

All samples, regardless of introduced cation, shows very high similar selectivity not dependent on the CO_2_ uptake. As is shown in Table 5, CO_2_ uptake in 273 K and 298 K decreases in the following order of dominating cation Li^+^ > Na^+^ > K^+^ > Ca^2+^ >Mg^2+^. This means that the L_i0.61_Na_0.39_X sample exhibits enhanced CO_2_ uptake with the preservation of very high selectivity, which is crucial for its potential application as a sorbent material.

## 3. Materials and Methods

### 3.1. Materials

A commercial zeolite 13X, supplied by Sigma-Aldrich, was chosen as a starting material in this work. Ion exchanged zeolites were prepared with the usage of 1.0 M aqueous solution of metal chlorides (Li^+^, K^+^, Mg^2+^, Ca^2+^). All the chloride salts of reagent grade were purchased from the commercial supplier POCH S.A., Poland. 

All gases were purchased from Air Liquide. The purity of all gases was 5.0, except for CO_2_, which had a purity of 4.5.

The following concentrated acids were used to dissolve zeolites: phosphoric, nitric, sulfuric and hydrofluoric. Each acid was reagent grade. 

The cation exchange was performed via a conventional method by exchanging ions between a liquid and a solid phase. A total of 2.0 g of sodium form of zeolite 13X was exchanged with 20 mL chloride solution of suitable cations (Li^+^, K^+^, Mg^2+^, Ca^2+^). All experiments were conducted for 2 h under constant agitation at 333–343 K. After this time, the samples were decanted and fresh solution was added. The procedure was repeated three times. Finally, the resulting samples were washed with distilled water and dried under vacuum at 473 K overnight.

The abovementioned procedure does not allow complete substitution of sodium cations, and thus, all of the presented results in this paper concern zeolites with a mixture of cations. Samples were named due to the degree of sodium exchange obtained from EDS analysis. For instance, sodium exchanged for lithium cations is named as follows: Li_y_Na_z_X, where ‘z’ stands for remaining quantity of sodium and ‘y’ is a quantity of introduced cation. In case of divalent cations, the quantity of introduced cation is halved due to the compensation of charge.

### 3.2. Methods

The textural properties of the ion-exchanged samples were determined by N_2_ adsorption/desorption isotherm measurements at 77 K using a Quadrasorb Si analyser (Quantachrome Instruments). Prior to analysis, the materials were degassed under vacuum at 473 K for 16 h. The specific surface areas were evaluated using the Brunauer-Emmet-Teller (BET) method. The total pore volumes (V_total_) were estimated at relative pressure P/P_0_ = 0.99. The pore size distribution (PSD) and micropores volume (V_micro_) were obtained from the Density Functional Theory (DFT) model in the Quantochrome software package. The submicropores volume (pores with size <0.8 nm) was calculated from CO_2_ adsorption at 273 K using the DFT method. 

X-ray diffraction patterns of the zeolite samples were acquired using an Empyrean diffractometer (PANalytical), equipped with a wide-angle detector (PIXcel) and a monochromator, which greatly lowered the signal to noise ratio. Data were collected over a 2θ range of 5–60° using copper radiation (λ_Kα1_ = 0.154056 nm). 

The ion-exchanged samples were also analyzed with scanning electron microscopy SEM SU8020 (Hitachi) coupled with energy-dispersive X-ray spectroscopy EDS NSS 312 (Thermo Scientific) allowing elemental microanalysis. The elemental microanalysis for other materials than polished surfaces is often considered semi quantitative. Therefore, variation of internal standard method was proposed and applied to measure the substitution degree of cations. The EDS results were confirmed with the ICP-OES method using an Optima 5300DV spectrometer (Perkin Elmer) and the ED-XRF method using an Epsilon 3 spectrometer (PanAnalytical). Prior to ICP analysis, each sample was added to a mixture of the following acids: nitric, sulfuric, phosphoric and hydrofluoric. Afterward, samples were placed in a microwave furnace for 20 min composed of four individual steps. The furnace was allowed to cool down to room temperature overnight. Furthermore, an additional sample was prepared containing only a mixture of acids, allowing to determine the influence of contamination originating from either acids or deionized water.

Gas analysis during selectivity measurement was performed using a mass spectrometer, quantitative gas analysis system QGA (Hiden Analytical). 

Carbon dioxide adsorption experiments were performed using a volumetric Quadrasorb apparatus (Quantachrome Instruments). Before the measurements, the samples were outgassed at 473 K under vacuum for 16 h. CO_2_ adsorption isotherms were determined at 273 K and 298 K up to a final pressure of 0.95 bar. 

Due to the very time-consuming measurement procedures, only the sample of the highest CO_2_ uptake was chosen to study its adsorption at different temperatures 298 K, 313 K, 333 K, 353 K and 373 K, up to a pressure of 1.8 bar. The measurements were performed using the Sievert’s apparatus (Hiden Isochema Intelligent Manometric Instrument—IMI). CO_2_ adsorption data on zeolites NaX and Li_0.61_Na_0.39_X were fitted to the Langmuir, Freundlich, Sips and Toth isotherm models. 

The Langmuir model assumes that the surface of adsorbent is homogeneous and the adsorption is localized in a monolayer. The Langmuir isotherm is expressed as Equation (2) [37,38]:

(2) $$q=\frac{q_{m}bp}{1+bp}$$

where q is the amount of carbon dioxide adsorbed per unit mass of zeolite (mmol/g), *p* is equilibrium pressure of CO_2_, *q_m_* is the maximum adsorption capacity (mmol/g) and *b* is a constant related to the adsorption energy.

The Freundlich isotherm is an empirical equation which is widely applied to describe heterogeneous systems. The Freundlich isotherm is expressed by the following Equation (3) [39]:

(3) $$q=k_{F}p^{n}$$

where k_F_ is the Freundlich constant and n is the heterogeneity factor. 

The Sips adsorption isotherm model is a combination of the Langmuir and Freundlich expression. The nonlinear Sips equation can be represented as Equation (4) [40]:

(4) $$q=\frac{q_{m}\;bp^{n}}{1+bp^{n}}$$

where *b* is the Langmuir constant and n is the Freundlich exponent. 

The Thot model is an empirical equation, which is very useful for describing heterogeneous systems and satisfies low and high-end boundary of the concentration. The Toth isotherm is expressed by the following Equation (5) [41]:

(5) $$q=\frac{q_{m}bp}{{\left[1+{\left(bp\right)}^{t}\right]}^{1/t}}$$

where *b* is the adsorption affinity and *t* is the Toth constant.

In this study, the nonlinear sum of the squares of the errors method was used to fit the experimental data, Equation (6) [42]:

(6) $$SSE=\overset{n}{\underset{i=1}{\sum }}{\left(q_{cal}-q_{exp}\right)}_{i}^{2}$$

where *q_cal_* is the calculated adsorbed quantity and *q_exp_* is the experimentally measured quantity.

The isosteric heat of adsorption was estimated using the Sips Equation (3) combined with the Clausius–Clapeyron Equation (7):

(7) $${\left(\frac{\delta ln\left(p\right)}{\delta \frac{1}{T}}\right)}_{\theta }=\frac{Q_{i}}{R}$$

where *ln(p)* is the natural logarithm of the pressure at specific surface loading (*θ*), *Qi* is the isosteric heat of adsorption, *T* is the temperature and *R* is the gas constant.

## 4. Conclusions

Sodium form of commercial zeolite 13X and its ion-exchanged forms, presented in this paper, are suitable for CO_2_ adsorption. Thorough analyses of these materials using ICP, EDS and XRF methods provided information about the level of the exchange. Taking into account that all three methods ended with similar results, we have concluded that the EDS method is much more useful, due to its ease and speed and because it avoids the need to use dangerous chemicals. 

The ion exchange led to textural and structural changes in a zeolite framework. The highest CO_2_ adsorption capacity was obtained for sample Li_0.61_Na_0.39_X. This sample revealed the highest specific surface area and the highest micropore volume. On the other hand, the lowest CO_2_ uptakes were observed for materials with introduced divalent cations, due to significantly decreased volume of micropores.

Moreover, introducing divalent cations into the zeolite framework increases volume of mesopores, which might be a result of relaxation of lattice strain introduced by the ion exchange.

The stability of obtained sorbents in multiple sorption/desorption aging cycles has been measured. The Li_0.61_Na_0.39_X sorbent exhibited very high stability during such measurements, which is crucial for application in industrial processes. An approximate 5% decrease of CO_2_ uptake was observed after 200 sorption/desorption cycles. As textural parameters do not change during the stability test, the changes in the surface chemistry, such as the formation of carbonates, must be responsible for observed decrease.

## Figures and Tables

**Table 1:** The sodium content of cation exchanged samples.

Sample	Remaining Na^+^ [%]		
	EDS	ICP	XRF
NaX	100	100	100
Li_0.61_Na_0.39_X	39	40	33
K_0.76_Na_0.24_X	24	18	13
Mg_0.32_Na_0.36_X	36	37	29
Ca_0.45_Na_0.10_X	10	10	6

**Table 2:** Textural properties of the investigated samples.

Sample	S_BET_ (m^2^/g)	V_total_ (cm^3^/g)	V_micro_ (cm^3^/g)	V_submicro_ (cm^3^/g)	V_meso_ (cm^3^/g)
L_i0.61_Na_0.39_X	624	0.35	0.35	0.27	0.01
NaX	569	0.32	0.30	0.24	0.01
K_0.76_Na_0.24_X	496	0.28	0.27	0.18	0.01
M_g0.32_Na_0.36_X	342	0.28	0.14	0.15	0.12
Ca_0.45_Na_0.10_X	431	0.30	0.20	0.17	0.08

**Table 3:** Ionic radii and lattice constants of the studied samples.

Sample	Cation Ionic Radius (Å) *	Lattice Constant (Å)
Li_0.61_Na_0.39_X	0.68	24.82
NaX	0.95	24.97
K_0.76_Na_0.24_X	1.33	25.08
Mg_0.32_Na_0.36_X	0.65	24.95
Ca0.45Na0.10X	0.99	24.90

* in ion-exchanged samples, the value of the cation radius stands for introduced cation.

**Table 4:** Integral breadth and calculated strains from the Wilson formula lattice.

Sample	Integral Breadth	Relative Lattice Strain (%)
NaX	0.169	-
Li_0.61_Na_0.39_X	0.177	0.43
K_0.76_Na_0.24_X	0.178	0.46
Mg_0.32_Na_0.36_X	0.189	0.70
Ca_0.45_Na_0.10_X	0.181	0.53

**Table 5:** CO_2_ uptake capacities of the studied samples.

Sample	CO_2_ Uptake (mmol/g)				
	273 K	298 K	298 K *	Pressure (Bar)	Reference
Li_0.61_Na_0.39_X	5.94	5.71	5.34	0.95	this study
NaX	5.55	5.45	5.10	0.95	this study
K_0.76_Na_0.24_X	4.54	4.26	-	0.95	this study
Mg_0.32_Na_0.36_X	3.19	2.64	-	0.95	this study
Ca_0.45_Na_0.10_X	3.58	3.18	-	0.95	this study
NaX	-	4.98	-	1.00	[21]
KX	-	4.44	-	1.00	[21]
LiX	-	5.62	-	1.00	[21]
Na-Zeolite β	-	2.80	-	1.00	[22]
SBA-15	-	0.50	-	1.00	[33]
Cs-MER	-	2.85	-	5.00	[34]
K-MER	-	4.20	-	5.00	[34]
Na-MER	-	4.80	-	5.00	[34]
13X	-	6.90	-	15.00	[35]
13X-K	-	6.90	-	20.00	[19]

* CO_2_ uptake after aging 200 adsorption/desorption cycles.

**Table 6:** Constant values of the Sips isotherms of CO_2_ adsorption on NaX.

T (K)	*q_m_* (mmol/g)	*b*	*n*
298	6.71	3.35	0.64
313	6.60	2.27	0.65
333	6.47	1.45	0.66
353	6.38	0.96	0.67
373	6.27	0.68	0.70

**Table 7:** Constant values of the Sips isotherms of CO_2_ adsorption on Li_0.61_Na_0.39_X.

T (K)	*q_m_* (mmol/g)	*b*	*n*
298	7.15	3.64	0.67
313	6.91	2.47	0.67
333	6.66	1.59	0.70
353	6.29	1.06	0.74
373	5.81	0.76	0.79

**Table 8:** Textural properties of the zeolites before and after aging tests.

Sample	S_BET_ (m^2^/g)	V_total_ (cm^3^/g)	V_micro_ (cm^3^/g)
Li_0.61_Na_0.39_X	624	0.35	0.35
L_i0.61_Na_0.39_X after 200 cycles	621	0.35	0.34
NaX	569	0.32	0.30
NaX after 200 cycles	579	0.32	0.30

**Table 9:** Selectivity of the samples.

Sample	CO_2_ Molar Fraction	Standard Deviation
Li_0.61_Na_0.39_X	0.97	0.01
NaX	0.96	0.02
K_0.76_Na_0.24_X	0.97	0.01
Mg_0.32_Na_0.36_X	0.96	0.03
Ca_0.45_Na_0.10_X	0.97	0.03

## Data Availability

The data presented in this article will be available upon request.

## Supplementary Materials

The following are available online, Figure S1: Results from mass spectrometer.

## Sample Availability

Samples of all used or obtained in this manuscript compounds are available in limited amount from the authors.

## References

[1] Gęsikiewicz-Puchalska A., Zgrzebnicki M., Michalkiewicz B., Narkiewicz U., Morawski A., Wrobel R. (2017). Improvement of CO_2_ uptake of activated carbons by treatment with mineral acids. Chem. Eng. J..

[2] Singh D., Croiset E., Douglas P., Douglas M. (2003). Techno-economic study of CO_2_ capture from an existing coal-fired power plant: MEA scrubbing vs. O_2_/CO_2_ recycle combustion. Energy Convers. Manag..

[3] Shindell D.T., Faluvegi G., Koch D.M., Schmidt G.A., Unger N., Bauer S.E. (2009). Improved attribution of climate forcing to emissions. Science.

[4] Bousquet P., Ciais P., Miller J.B., Dlugokencky E.J., Hauglustaine D.A., Prigent C., van der Werf G.R., Peylin P., Brunke E.G., Carouge C. (2006). Contribution of anthropogenic and natural sources to atmospheric methane variability. Nature.

[5] Chiesa P., Consonni S. (1999). Shift reactors and physical absorption for low-CO_2_ emission IGCCs. J. Eng. Gas. Turbines Power.

[6] Chue K.T., Kim J.N., Yoo Y.J., Cho S.H., Yang R.T. (1995). Comparison of activated carbon and zeolite 13X for CO_2_ recovery from flue gas by pressure swing adsorption. Ind. Eng. Chem. Res..

[7] Cornelissen R., Hirs G. (1998). Exergy analysis of cryogenic air separation. Energy Convers. Manag..

[8] Powell C.E., Qiao G. (2006). Polymeric CO_2_/N_2_ gas separation membranes for the capture of carbon dioxide from power plant flue gases. J. Membr. Sci..

[9] Ho M.T., Allinson G.W., Wiley D.E. (2008). Reducing the cost of CO_2_ capture from flue gases using pressure swing adsorption. Ind. Eng. Chem. Res..

[10] Sayari A., Belmabkhout Y., Serna-Guerrero R. (2011). Flue gas treatment via CO_2_ adsorption. Chem. Eng. J..

[11] Hinkov I., Lamari F.D., Langlois P., Dicko M., Chilev C., Pentchev I. (2016). Carbon dioxide capture by adsorption. J. Chem. Technol. Metall..

[12] Siriwardane R.V., Shen M.-S., Fisher E.P., Poston J.A. (2001). Adsorption of CO_2_ on molecular sieves and activated carbon. Energy Fuels.

[13] Choi S., Drese J.H., Jones C.W. (2009). Adsorbent materials for carbon dioxide capture from large Anthropogenic point sources. ChemSusChem.

[14] Flanigen E.M., Jansen J.C., van Bekkum H. (1991). Introduction to Zeolite Science and Practice.

[15] Panneerselvam P., Thinakaran N., Thiruvenkataravi K., Palanichamy M., Sivanesan S. (2008). Phosphoric acid modified-Y zeolites: A novel, efficient and versatile ion exchanger. J. Hazard. Mater..

[16] Harlick P.J., Tezel F.H. (2004). An experimental adsorbent screening study for CO_2_ removal from N2. Microporous Mesoporous Mater..

[17] Siriwardane R.V., Shen A.M.-S., Fisher E.P., Losch J. (2005). Adsorption of CO_2_ on Zeolites at moderate temperatures. Energy Fuels.

[18] Sarker A.I., Aroonwilas A., Veawab A. (2017). Equilibrium and kinetic behaviour of CO2 adsorption onto Zeolites, Carbon Molecular Sieve and Activated Carbons. Energy Procedia.

[19] Garshasbi V., Jahangiri M., Anbia M. (2017). Equilibrium CO_2_ adsorption on zeolite 13X prepared from natural clays. Appl. Surf. Sci..

[20] Cavenati S., Grande A.C.A., Rodrigues A.E. (2004). Adsorption equilibrium of Methane, Carbon Dioxide, and Nitrogen on Zeolite 13X at high pressures. J. Chem. Eng. Data.

[21] Walton K.S., Abney M.B., LeVan M.D. (2006). CO_2_ adsorption in Y and X zeolites modified by alkali metal cation exchange. Microporous Mesoporous Mater..

[22] Yang S.-T., Kim J., Ahn W.-S. (2010). CO_2_ adsorption over ion-exchanged zeolite beta with alkali and alkaline earth metal ions. Microporous Mesoporous Mater..

[23] Chen C., Park D.-W., Ahn W.-S. (2014). CO_2_ capture using zeolite 13X prepared from bentonite. Appl. Surf. Sci..

[24] Mulgundmath V.P., Tezel F.H., Saatcioglu T., Golden T.C. (2012). Adsorption and separation of CO_2_/N_2_ and CO_2_/CH_4_ by 13X zeolite. Can. J. Chem. Eng..

[25] Hovington P., Timoshevskii V., Burgess S., Demers H., Statham P., Gauvin R., Zaghib K. (2016). Can we detect Li K X-ray in lithium compounds using energy dispersive spectroscopy?. Scanning.

[26] Ravikovitch P.I., Neimark A. (2001). Characterization of nanoporous materials from adsorption and desorption isotherms. Colloids Surf. A Physicochem. Eng. Asp..

[27] Treacy M.M.J., Higgins J.B. (2007). Collection of Simulated XRD Powder Patterns for Zeolites.

[28] Baerlocher C., McCusker L., Olson D. (2007). Atlas of Zeolite Framework Types.

[29] Klug H., Alexander L. (1974). X-ray Diffraction Procedures: For Polycrystalline and Amorphous Materials.

[30] Presser V., McDonough J., Yeon S.-H., Gogotsi Y. (2011). Effect of pore size on carbon dioxide sorption by carbide derived carbon. Energy Environ. Sci..

[31] Wei H., Deng S., Hu B., Chen Z., Wang B., Huang J., Yu G. (2012). Granular bamboo-derived activated Carbon for high CO2Adsorption: The dominant role of narrow micropores. ChemSusChem.

[32] Serafin J., Narkiewicz U., Morawski A.W., Wróbel R.J., Michalkiewicz B. (2017). Highly microporous activated carbons from biomass for CO_2_ capture and effective micropores at different conditions. J. CO_2_ Util..

[33] Liu X., Li J., Zhou L., Huang D., Zhou Y. (2005). Adsorption of CO_2_, CH_4_ and N_2_ on ordered mesoporous silica molecular sieve. Chem. Phys. Lett..

[34] Georgieva V.M., Bruce E.L., Verbraeken M.C., Scott A.R., Casteel W.J., Brandani S., Wright P.A. (2019). Triggered gate opening and breathing effects during selective CO_2_ adsorption by Merlinoite Zeolite. J. Am. Chem. Soc..

[35] Liang Z., Marshall M., Chaffee A.L. (2009). CO2 adsorption-based separation by metal organic framework (Cu-BTC) versus Zeolite (13X). Energy Fuels.

[36] Bertsch L., Habgood H.W. (1963). An infrared spectroscopic study of the adsorption of water and carbon dioxide by Linde Molecular Sieve X1. J. Phys. Chem..

[37] Sreńscek-Nazzal J., Narkiewicz U., Morawski A.W., Wróbel R.J., Michalkiewicz B. (2015). Comparison of optimized Isotherm models and error functions for Carbon Dioxide adsorption on Activated Carbon. J. Chem. Eng. Data.

[38] Foo K.Y., Hameed B.H. (2010). Insights into the modeling of adsorption isotherm systems. Chem. Eng. J..

[39] Hameed B., Mahmoud D., Ahmad A.L. (2008). Equilibrium modeling and kinetic studies on the adsorption of basic dye by a low-cost adsorbent: Coconut (Cocos nucifera) bunch waste. J. Hazard. Mater..

[40] Salmasi M., Fatemi S., Rad M.D., Jadidi F. (2013). Study of carbon dioxide and methane equilibrium adsorption on silicoaluminophosphate-34 zeotype and T-type zeolite as adsorbent. Int. J. Environ. Sci. Technol..

[41] Behvandi A., Tourani S. (2011). Equilibrium modeling of carbon dioxide adsorption on zeolites. Int. Sch. Sci. Res. Innov..

[42] Porter J., McKay G., Choy K. (1999). The prediction of sorption from a binary mixture of acidic dyes using single- and mixed-isotherm variants of the ideal adsorbed solute theory. Chem. Eng. Sci..

