# Supplementary material for: Changes in Porous Parameters of the Ion Exchanged X Zeolite and Their Effect on CO2 Adsorption
Source: Molecules. 2021 Dec 11;26(24):7520. doi: 10.3390/molecules26247520 (PMC8707879; doi:10.3390/molecules26247520)
Supplement: Supplementary file 1 [file molecules-26-07520-s001.zip › molecules-1475281-supplementary.pdf]

Supporting Materials

Changes in porous parameters of the ion exchanged X zeolite and their effect on CO_2 adsorption

Andżelika Gęsikiewicz-Puchalska [1], Michal Zgrzebnicki [1], Beata Michalkiewicz [1], Agnieszka Kałamaga [1], Urszula Narkiewicz [2], Antoni W. Morawski [2] and Rafal Wrobel [1,*]

West Pomeranian University of Technology, Szczecin, Faculty of Chemical Technology and Engineering
1 Department of Catalytic and Sorbent Materials Engineering
2 Department of Inorganic Chemical Technology and Environment Engineering
* Correspondence: rafal.wrobel@zut.edu.pl

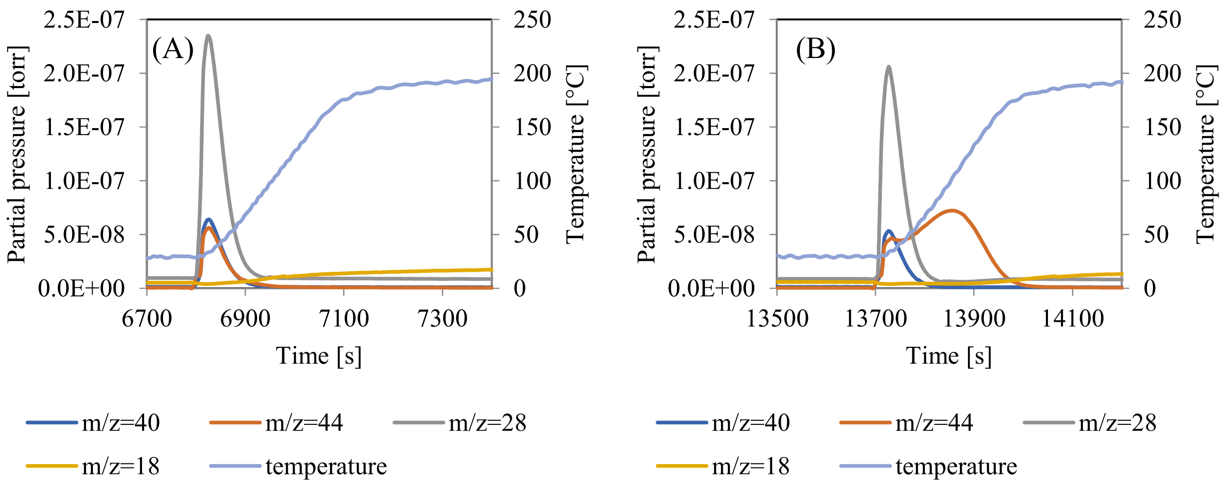

Figure S1. Results from mass spectrometer.
